# Buffering deleterious polymorphisms in highly constrained parts of HIV-1 envelope by flexible regions

**DOI:** 10.1186/s12977-016-0285-6

**Published:** 2016-07-30

**Authors:** Romain Gasser, Meriem Hamoudi, Martina Pellicciotta, Zhicheng Zhou, Clara Visdeloup, Philippe Colin, Martine Braibant, Bernard Lagane, Matteo Negroni

**Affiliations:** 1 Architecture et Réactivité de l’ARN, Université de Strasbourg, CNRS, IBMC, 15 rue René Descartes, 67084 Strasbourg, France; 2 U1016, UMR 8104, INSERM-CNRS, Institut Cochin, Université Paris Descartes, Paris, France; 3 INSERM U1108, Viral Pathogenesis Unit, Department of Virology, Institut Pasteur, Paris, France; 4 INSERM U966, Université François Rabelais, Tours, France

**Keywords:** HIV, Envelope, Coevolution, Viral entry, Antigenic variation

## Abstract

**Background:**

Covariation is an essential process that leads to coevolution of parts of proteins and genomes. In organisms subject to strong selective pressure, coevolution is central to keep the balance between the opposite requirements of antigenic variation and retention of functionality. Being the viral component most exposed to the external environment, the HIV-1 glycoprotein gp120 constitutes the main target of the immune response. Accordingly its more external portions are characterised by extensive sequence heterogeneity fostering constant antigenic variation.

**Results:**

We report that a single polymorphism, present at the level of the viral population in the conserved internal region C2, was sufficient to totally abolish Env functionality when introduced in an exogenous genetic context. The prominent defect of the non-functional protein is a block occurring after recognition of the co-receptor CCR5, likely due to an interference with the subsequent conformational changes that lead to membrane fusion. We also report that the presence of compensatory polymorphisms at the level of the external and hypervariable region V3 fully restored the functionality of the protein. The functional revertant presents different antigenic profiles and sensitivity to the entry inhibitor TAK 779.

**Conclusions:**

Our data suggest that variable regions, besides harbouring intrinsic extensive antigenic diversity, can also contribute to sequence diversification in more structurally constrained parts of the gp120 by buffering the deleterious effect of polymorphisms, further increasing the genetic flexibility of the protein and the antigenic repertoire of the viral population.

## Background

Entry of the human immunodeficiency virus type 1 (HIV-1) into the target cell begins with the recognition by the viral envelope (Env) of the receptor CD4, a molecule of the immunoglobulin superfamily [1]. This step is followed by conformational changes of the Env protein [2] that generate its co-receptor binding site, in most cases the member of the G protein-coupled chemokine receptors CCR5, with a tendency to shift to the use of CXCR4 during the late phases of the acquired immunodeficiency syndrome [3, 4]. This binding triggers further structural rearrangements, leading to a conformation competent for promoting fusion of the viral and cellular membranes [5]. Progressive stepwise unmasking of different epitopes that occurs during the process implies that most of the conformations adopted by the protein are created only once the target cell has been recognised and, thereby, in a confined environment where the immune system has limited access. Despite this, recognition of Env by the immune system is manifestly the crucial immunological parameter for the control of viral replication, Env being the only target of neutralizing immune response documented to date [6–9].

The extensive genetic diversity of HIV-1 Env therefore constitutes a main asset for viral escape to immune response. However, genetic diversity in a protein that must undergo such extensive conformational changes constitutes an intrinsic weakness since each genetic polymorphism must be simultaneously compatible with all the various architectural arrangements. Indeed, relentless sequence variation due to misincorporation occurring during reverse transcription [10], to hypermutagenesis by cellular restriction factors [11], and to pervasive recombination occurring throughout the HIV genome [12–14] constantly challenges retention of functionality. And these requirements restrict the sequence space the protein can explore, limiting its antigenic diversity [15, 16]. Extensive coevolution of structurally and functionally related parts of the protein is the solution for the virus to conciliate genetic diversity and maintain of functionality.

In particular, deciphering coevolution networks in the gp120 is important at least from three standpoints. One is to provide insights into the dynamics of protein evolution in a model organism that undergoes physiological extensive sequence diversity. Second, it is important for understanding the tridimensional arrangement of the protein. A number of structures of HIV-1 Env has been indeed obtained under different conformations, mostly pre-CD4 bound [17, 18] and CD4 bound [19]. The remarkable structural flexibility of the protein, though, requires the use either of ligands (as antibodies) or of mutated or truncated versions of the protein to get a complex sufficiently rigid as to allow structural studies [5, 17–19]. In particular, mutations of the variable regions of the protein are frequently employed [20, 21]. All these modifications of the native protein can alter the natural conformation of the protein or stabilize it in conformations that might not be fully representative of those most commonly assumed by the free protein [22]. Furthermore, the arrangements of the protein during the recognition of the coreceptor and the subsequent conformational rearrangements triggered by this interaction still remains to be understood. For this step of the entry process, the only structure available to date is a soluble CD4-gp120 monomer bound to the tyrosine-sulfated antibody 412d that mimics CCR5 binding. This structure has allowed docking the N-terminal portion of CCR5 at the base of the V3 loop [23]. The second extracellular portion of the coreceptor, instead, has been mapped, by mutational analysis, to interact with the apical loop of V3 [24]. A structure of the Env trimer complexed to CD4 and CCR5, which would define the architecture of a prefusion intermediate, is however not yet available. Finally, understanding coevolution networks can guide the design of antigenic modifications while retaining its functionality, an important issue for vaccine purposes. Properly folded antigens are indeed essential for eliciting neutralizing antibodies and the presence on VLP used for vaccines of a high proportion of non-correctly folded trimers appears as an important factor limiting the efficiency of vaccine approaches [25, 26].

As mentioned above, recombination is pervasive during HIV-1 replication and it is a process particularly challenging for the preservation of functionality in the presence of extensive coevolution networks. Indeed, recombination leads, in a single replication cycle, to the generation of multiple polymorphisms, often requiring the rapid introduction of several compensatory mutations to retain functionality. Accordingly, previous studies demonstrated the fragility of various HIV-1 genes to functional inactivation by recombination [16, 27, 28]. Starting from these observations, we recently probed the functionality of Env after swapping the variable regions V1V2 that, together with V3, are the most external portions of the protein in the pre-CD4 bound state. We observed that substituting these hypervariable domains from one primary isolate with that of another isolate was responsible for a dramatic drop in functionality in the chimerical protein. This indicates that also these parts of the protein, which are located in peripheral positions and harbour extensive genetic diversity, are submitted to coevolution constraints [29]. These results underlined the poor robustness of HIV-1 Env despite its well-known sequence diversity.

In the present work, through the initial generation of chimerical proteins, as a model of recombination, we focus on the involvement in coevolution of the constant region C2, a part of the protein central to several steps of the process of viral entry. This region not only is crucial because it constitutes the link between the two main domains of the gp120 (inner and outer domain) and contributes to the formation of essential structures as the bridging sheet, but also because it is located in a central portion of the Env core. Sequence diversification in this region can therefore have dramatic consequences on the global arrangement of the protein. Furthermore, it has been shown recently that C2 participates in inducing neutralising antibodies as 8ANC195 [30–32]. We report here, that genetic variation in a variable region of the protein such as V3 can relieve a dramatic decrease in functionality induced by a single sequence polymorphism in a constant region as C2. This underlines the importance of flexible regions of the gp120 in extending the sequence space that can be explored by this antigen, central for neutralisation of HIV-1.

## Results

### Replacement of the constant region C2 abolishes Env functionality

**Fig. 1:**
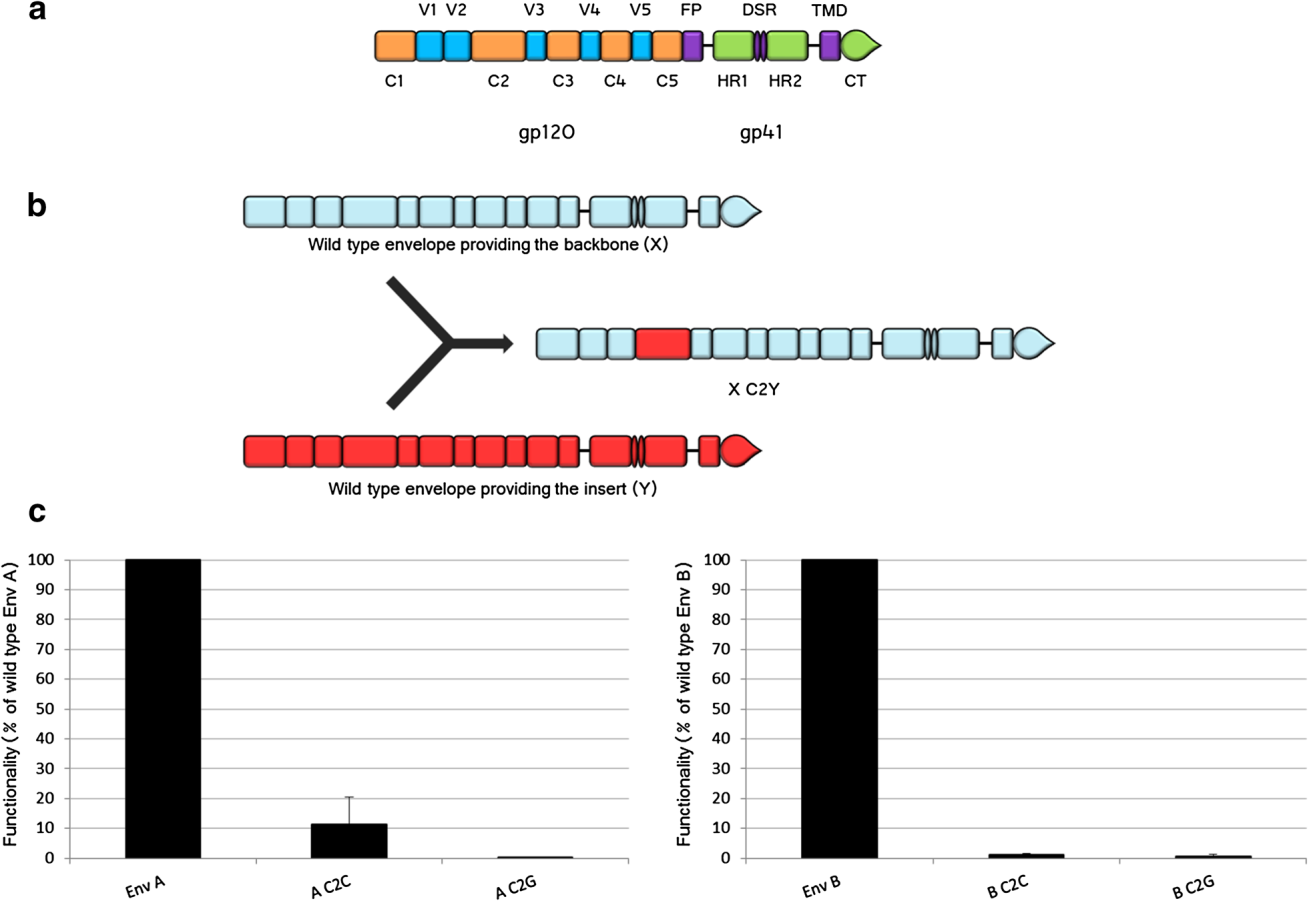
Interference of C2 phylogenetic origin with Env functionality. **a** Representation of the different domains of HIV Env. gp120: *orange* for constant (C1–C5) and *blue* for variable (V1–V5) regions; for gp41 the different domains are *green* and *purple*. *FP* fusion peptide, *HR1 and HR2* heptad repeats 1 and 2, respectively, *DSR* disulphide bonded region, *TMD* trans-membrane domain, *CT* cytoplasmic tail. **b** Principle of construction of C2 chimeras. The Env providing the backbone (X) is in *pale blue*, the one providing the insert in *red* (Y). The resulting chimera is named X C2Y. **c** Levels of viral entry of vectors carrying a reporter luciferase gene and having a chimerical C2 Env are shown as percentage of the infectivity observed with the corresponding wild type Env. For each independent experiment the percentage of functionality was determined with respect to the corresponding wild type Env, which was run in parallel, and the values used for the graph correspond to the average of these percentages with *error bars* indicating the standard deviation. n varies between 3 and 9, depending on the sample considered. *Left panel* chimeras with an Env A backbone, *right panel* chimeras with a B Env backbone

To study the involvement of the constant region C2 in coevolution with other portions of HIV-1 Env, we have generated four chimeric envelopes starting from primary isolates of different subtypes of HIV-1 group M. The isolates used were chosen on the basis of previous works where we observed a loss of functionality for recombinant and chimeric Env generated starting from these envelopes [28, 29]. In an Env protein from either an isolate of subtype A or B (called “Env A” and “Env B”, respectively) we have replaced the original C2 region with the C2 from an isolate of either group C or group G (whose envelopes will be referred as “Env C” and “Env G”, respectively). The resulting chimeras have been called A C2C and A C2G, for the Env A protein that contains the C2 region either from Env C, or from Env G, respectively (Fig. 1a, b). Similarly, Env B proteins that have been modified to contain the C2 region from Env C or from Env G, are named B C2C and B C2G, respectively (in order to avoid confusion we will always refer to amino acids using the three-letter code and single capital letters will always refer to HIV-1 M subtypes). The rationale for the choice of these specific chimeras was that, since we were searching for a possible perturbation of coevolution networks that would translate into a decrease in functionality, such a decrease would have been more manifest if the insertion of the exogenous sequence was made using as backbones the most functional isolates, in our case isolates A and B [28, 29].

The functionality of the chimerical envelopes has been tested by viral entry assays, as described in “Methods” section. For this test, pNL4-3.Luc.E^−^ virions complemented by the individual chimeric envelopes to test have been used. All four chimeras displayed a dramatic drop in functionality with a residual functionality below 1 % in three cases and around 10 % for the chimera A C2C (Fig. 1c). Therefore, amino acids present in the C2 regions of isolates C and G, which were inserted in the A or B envelopes, dramatically perturb the functionality of both proteins.

**Fig. 2:**
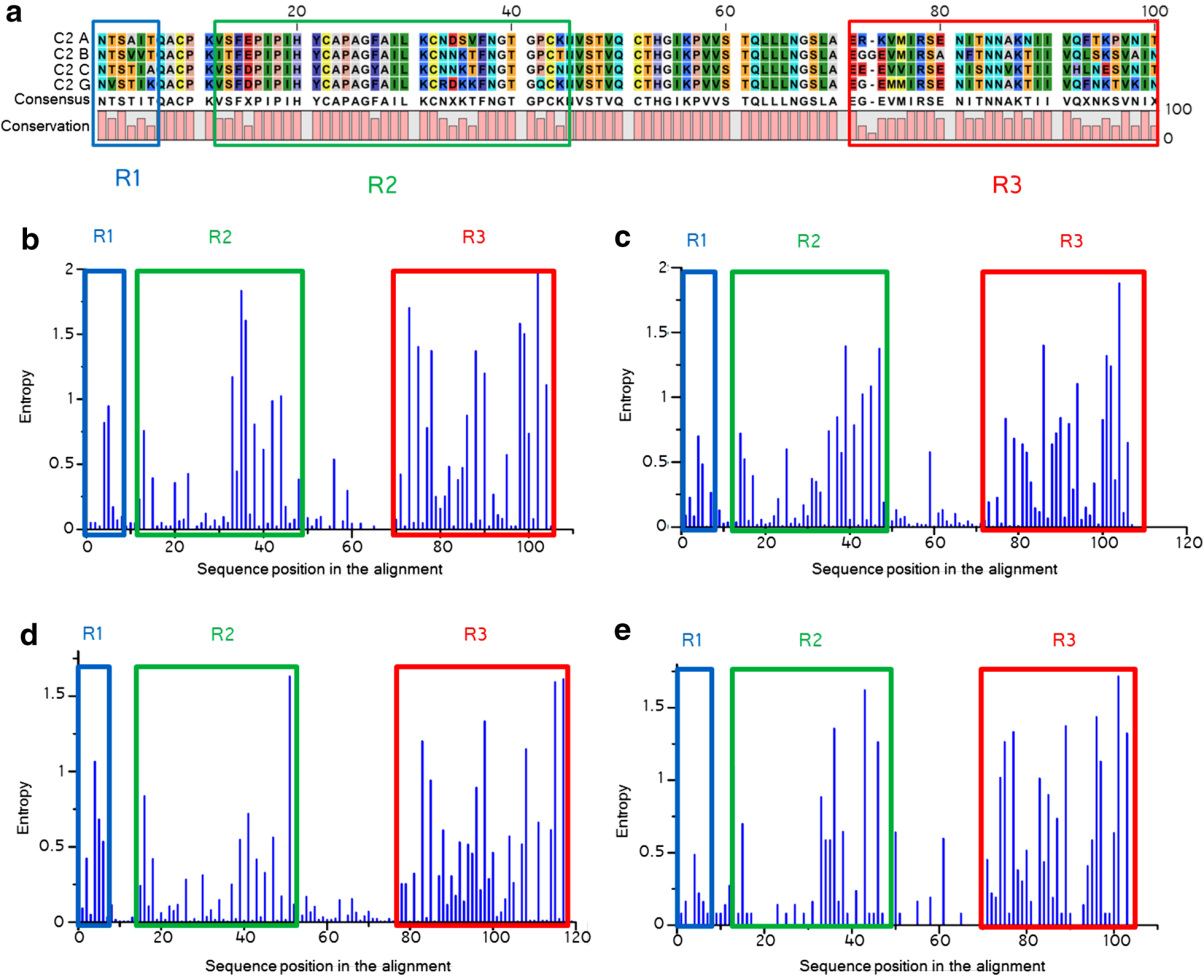
Alignments and entropy plots in Env sequences from the database for the C2 region. **a** Sequence alignment of the region C2 for the four wild type Env proteins used in this study. Color code: *orange* hydrophilic, *green* hydrophobic, *red* acidic, *light blue* weak basic, *blue* basic, *violet* aromatic, *light violet* histidine, *yellow* sulphur-containing, *white* glycine, *gray* alanine, *pink* proline. The three variable sub-regions we defined are highlighted by *squares*: R1 in *blue*, R2 in *green* and R3 in *red*. **b**–**e** Shannon entropy plots. Data were obtained from 201 isolates for subtype A1 (**b**), 1719 isolates for subtype B (**c**), 1361 isolates for subtype C (**d**), and 63 isolates for subtype G (**e**). The three variable sub-regions are delimited using the *same color* code as in **a**. The limits taken to define the borders of the C2, R1, R2, and R3 regions in the different isolates are the following, according to HxB2 numbering: 197–296 (included) for C2, 197–202 for R1, 208–240 for R2, 268–296 for R3. The slightly different sizes of the regions in the different subtypes are due to the presence of insertions and deletions in the multiple sequence alignments in the data from the database

In order to identify the determinants for the drop in functionality caused by the insertion of the exogenous C2, we aligned the C2 region from the four isolates used. Sequence variability was circumscribed to three areas that we named R1, R2 and R3, for region 1, 2, and 3, respectively (Fig. 2a). Clustering of sequence diversity in these three regions is not limited to our isolates but well recaptures the pattern of sequence divergence at the population level, as shown by Shannon entropy analysis along Env performed on isolates issued from the database for each of the four subtypes used in the study (Fig. 2b–e). This underscores the pertinence at the level of the viral population of the isolates employed in the present study.

### Identification of the region responsible for the loss of functionality in the C2 chimeras

**Fig. 3:**
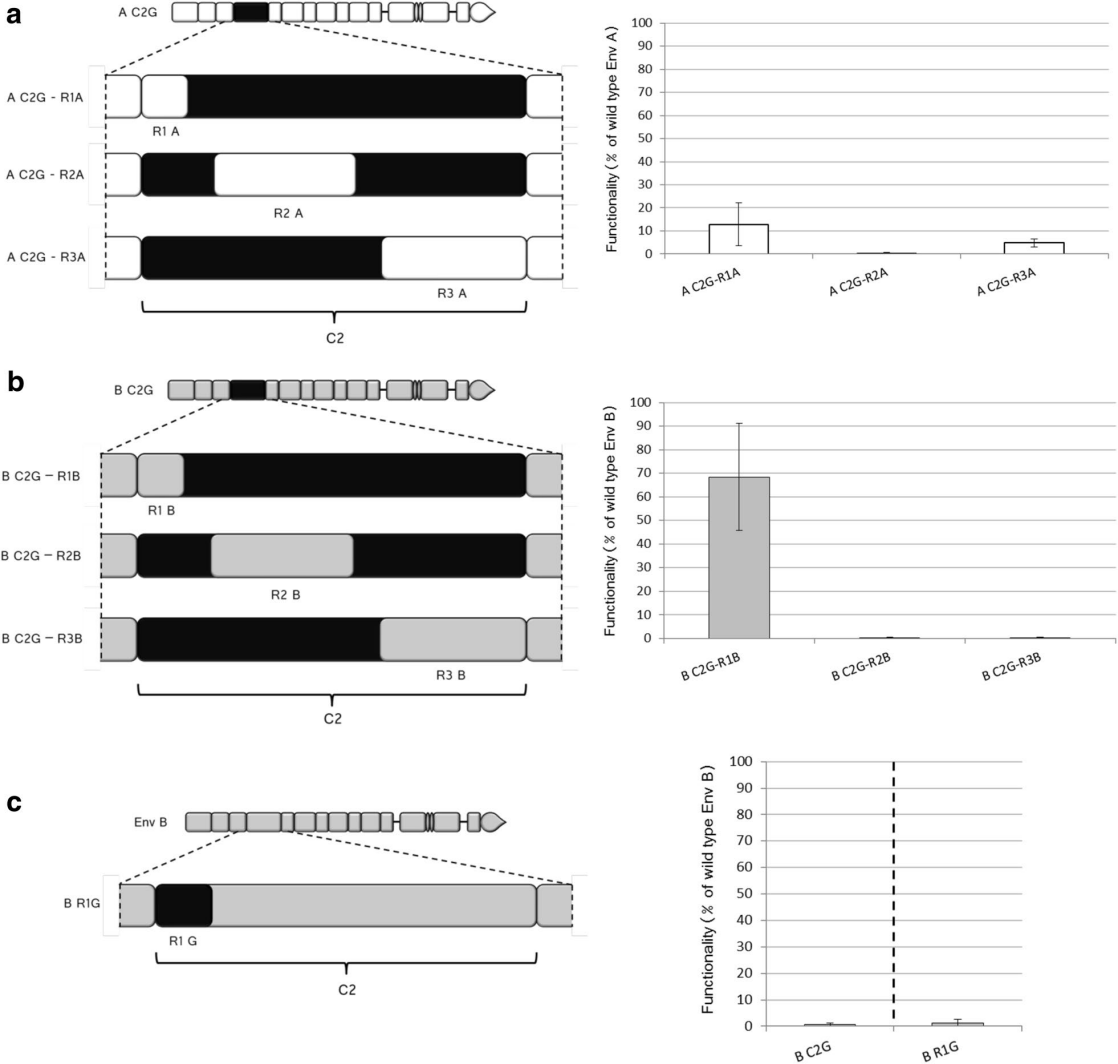
Involvement of the three variable sub-regions of C2 in the modulation of Env functionality. In each *panel*, a schematic representation of the chimerical Env is provided on the *left* and the level of functionality on the *right*. Color code: Env A *white*, Env B *grey*, env G *black*. **a**, **b** Search for restoration of functionality starting from C2 chimeras. **a** Envelopes generated starting from the A C2G chimera by replacement of R1, R2, and R3 from isolate A. Percentages of functionality were calculated as for Fig. 1. n varies between 3 and 9, depending on the sample considered. **b** Envelopes generated starting from the B C2G chimera by replacement of R1, R2, and R3 from isolate B. n varies between 3 and 9, depending on the sample considered. **c** Env generated by inserting in wt B Env the sequence R1 from isolate G. The functionality of the B C2G chimera is given as reference on the left of the *dotted line*. n varies between 4 and 9, according to the sample considered

The loss of functionality observed in the chimeras is necessarily due to the replacement of amino acids in the C2 region present in the original A or B sequences by those present in isolates C or G. Since the differences among these isolates were confined to regions R1, R2, and R3, the amino acids that cause the drop in functionality of the chimeras must be located either in one of these regions or in a combination of them. To assess their relative contribution to the drop in functionality observed, we focused on the A C2G and B C2G chimeras (since the insertion of the C2 sequence from isolate G triggered a stronger drop in functionality than that of C2 from isolate C, Fig. 1c) and we investigated at which extent inserting R1, R2, or R3 from isolate A or B would restore the functionality of the chimera (Fig. 3, panels a and b, respectively). Re-insertion of the original sequence R1 restored functionality at around 13 % for Env A (Fig. 3a) and 70 % for Env B (Fig. 3b). Both increases are significant (p < 0.05 and p < 0.001, respectively), indicating that these regions contain important determinants for the loss of functionality observed in the A C2G and B C2G chimeras. Replacement of R2, instead, did not lead to detectable recovery neither for the A nor for the B backbones (p values, 0.384 and 0.152, respectively). Finally, the replacement of R3 led to a modest recovery (5 % of functionality, p < 0.05) for isolate A while no effect was observed for isolate B (p = 0.488).

Altogether, the clearest picture emerges from the results obtained with the B Env for which the R1 sequence alone restores the functionality of the originally defective B C2G chimera to 70 % of the functionality of wt B Env. This indirectly suggests that this region harbours most determinants for the drop of functionality of B C2G with respect to wt B Env. To confirm this interpretation, we generated a wt Env B where we replaced uniquely the R1 region by that of the G Env (B R1G chimera, Fig. 3c). The presence of the exogenous R1 sequence alone was sufficient to drop functionality to levels comparable to those obtained for the replacement of the whole C2 region (Fig. 3c). This indicates that the sequence polymorphisms differentiating B and G envelopes in R1 are sufficient to account for an almost complete drop in functionality of the B Env.

### The polymorphysm at position 202 induces the loss of functionality

**Fig. 4:**
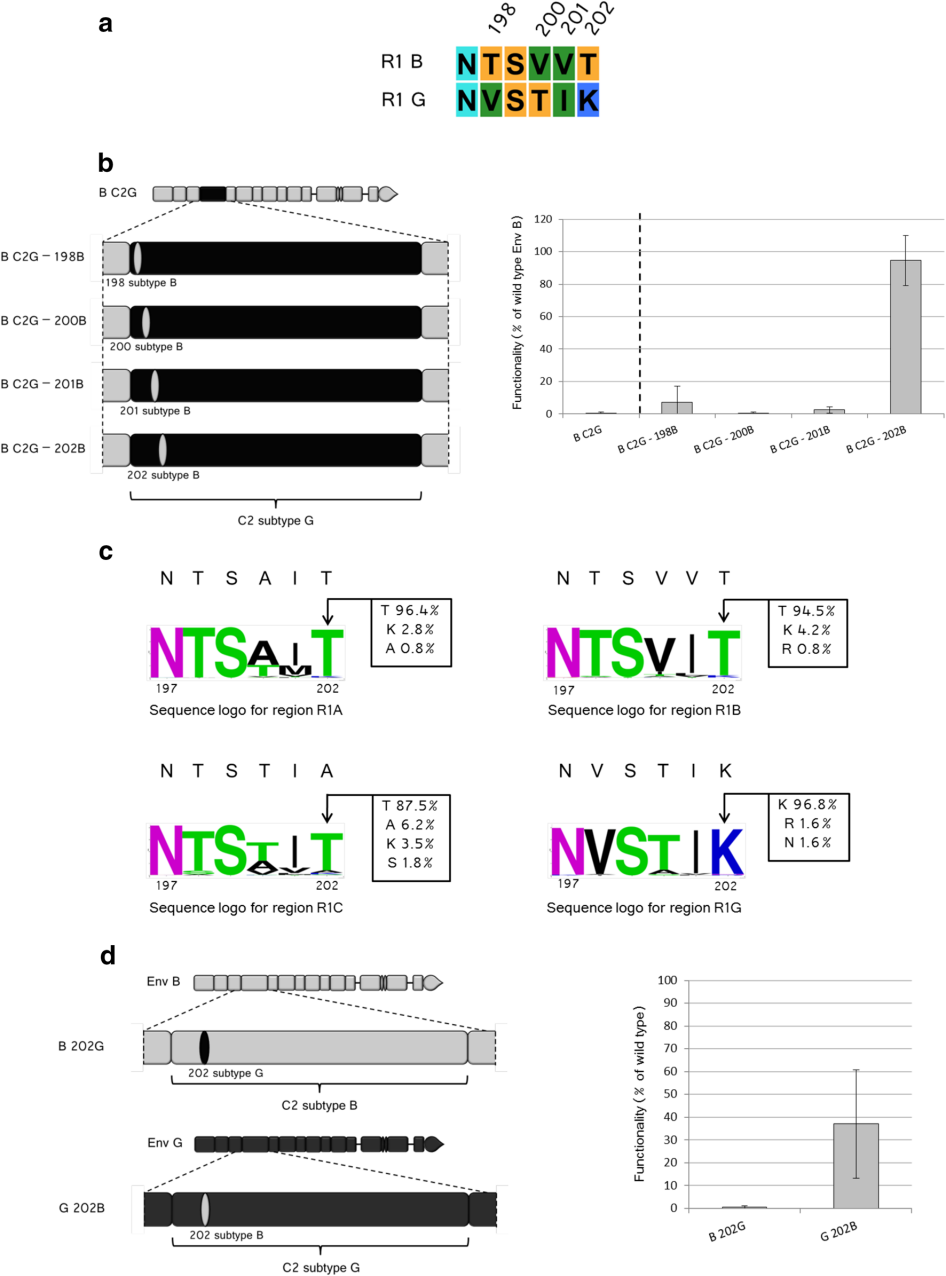
Identification of the amino acid responsible for the loss of functionality in R1 chimeras. **a** Sequence alignment between regions R1 of the Env B and G showing the four amino acids differing between the two sequences: positions 198, 200, 201 and 202 (numbering according to HxB2). Color code is the same as for Fig. 2a. **b** *Left* Schematic representation of the B C2G chimeras carrying the substitutions of the individual amino acids indicated. Color code: Env B *grey*, Env G *black*. *Right* Functionality of the Env depicted on the *left*. The functionality of B C2G is given as reference on the *left* of the *dotted line*. n varies between 4 and 9, according to the sample considered. **c** Sequence logo (WebLogo, http://weblogo.berkeley.edu/logo.cgi) for R1 sub-regions of different subtypes are shown, subtype A at *top left*, subtype B *top right*, subtype C *bottom left* and subtype G *bottom right*; the relative prevalence at the position equivalent to position 202 is indicated. The sequence of the isolates used in our study is given on the *top* of each sequence logo. Color code: *purple* weakly basic residues, *green* polar residues, *black* hydrophobic residues, *blue* basic residues. **d** Schematic representation (*left*) and level of functionality (*right*) of Env B 202G and of Env G 202B. Percentages of functionality were calculated as for Fig. 1. n varies between 6 and 9, depending on the sample considered

Despite the dramatic difference at the level of functionality, wt B and B R1G Env differ only for the four amino acids shown in Fig. 4a. To evaluate their specific involvement, we checked whether the replacement in B R1G Env of each individual amino acid by those present in the B Env would restore functionality (Fig. 4b). The replacement from Lys to the original Thr present in wt B Env at position 202 was sufficient to fully restore functionality of B R1G bringing it to levels no longer significantly different from those of wt B Env (p = 0.584). Replacement at the other three positions, instead, did not significantly alter functionality with respect to the starting chimera (p values: 0.328, 0.766 and 0.082 for positions 198, 200, and 201, respectively). At the level of the viral population, two of the four positions that vary among the four subtypes considered in R1 (positions 200 and 201) are poorly conserved, particularly in subtypes A and C (Fig. 4c), reflecting genetic flexibility of these sites. This can account for the lack of restoration of functionality observed when replacing residues in these two positions. The other two positions (198 and 202), in contrast, presented a high subtype-specific conservation, with subtype G deviating from the other subtypes (Val in place of Thr for position 198 and Lys instead of Thr for position 202). This suggests that these amino acids could have coevolved with other subtype-specific residues elsewhere in the protein. Concerning residue 202, the restoration of functionality observed by inserting in this position the amino acid of the same phylogenetic origin than the regions other than C2 suggests that its coevolving partner is located outside C2. In contrast, the fact that replacement at position 198 by the amino acid of the same origin than the regions outside C2 failed to restore functionality suggests that its coevolving partner might instead be located within C2.

To confirm the importance of position 202, we then made the “mirror” replacement by substituting, in wt B Env, uniquely the amino acid at position 202 by the one present in isolate G (Lys instead of Thr), generating B 202G Env. The functionality of this Env was nearly undetectable (Fig. 4d) confirming the central role of this polymorphism for modulating Env functionality. Since position 202 of isolate G is incompatible with the rest of the Env B backbone it would also be expected that the reversal situation (the presence at position 202 of the Thr from Env B instead of the Lys of Env G) would perturb the functionality of Env G. Indeed, a significant loss of functionality (p < 0.01) was observed also in this case (Fig. 4d), although significantly less severe (p < 0.05) than in the case residue 202 from Env G inserted in Env B.

### The coevolving partners of residue 202: identification of the region

**Fig. 5:**
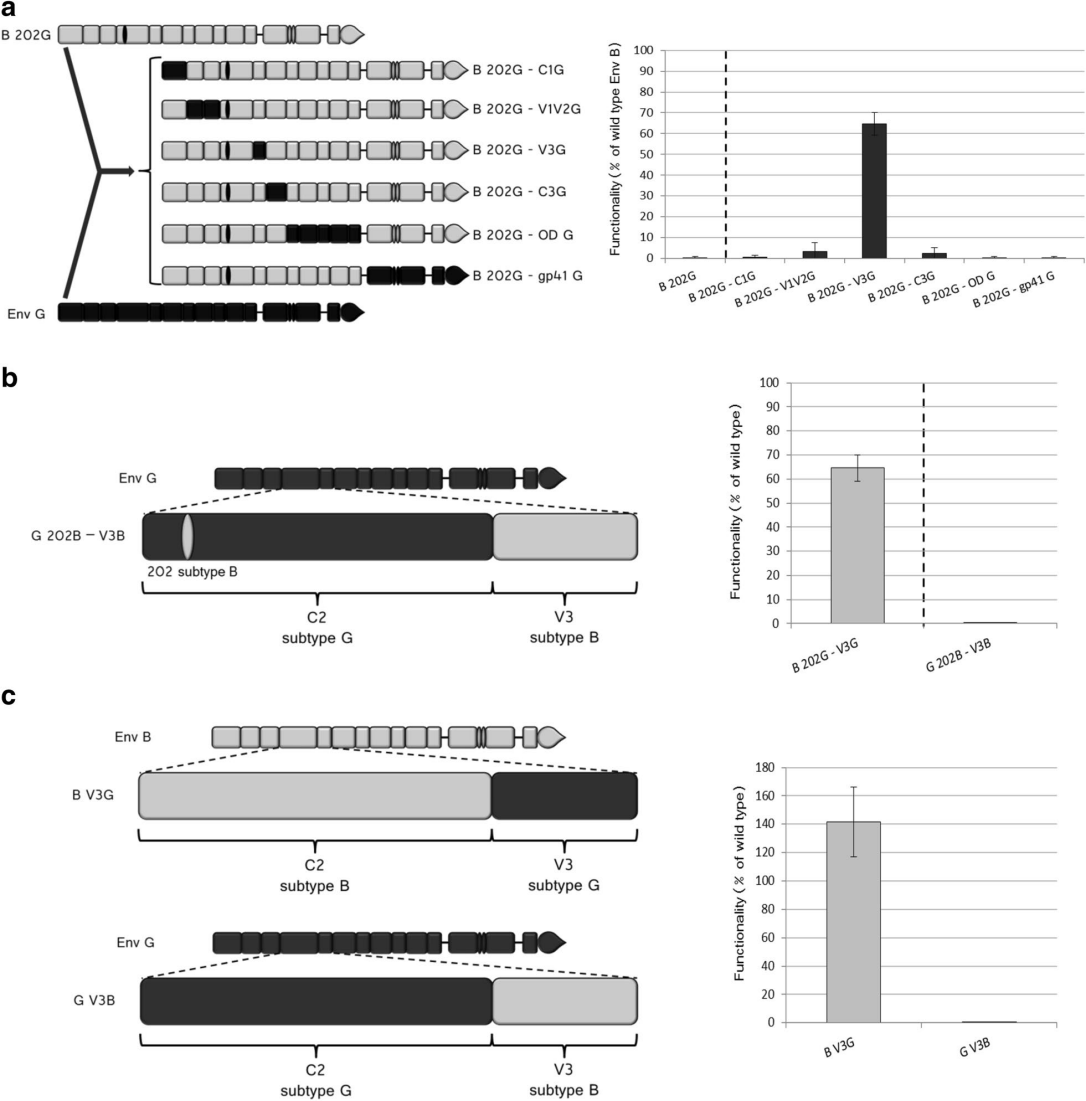
Identification of the coevolving partner of amino acid 202. In each *panel*, a schematic representation of the chimerical Env is provided on the *left* and the level of functionality on the *right*. Color code: Env B *grey*, env G *black*. **a** Various regions of Env G are replaced in Env B 202G, as indicated. The functionality of the Env B 202G is given as reference on the *left* of the *dotted line*. n varies between 3 and 6, according to the sample considered. **b** Functionality of G 202G Env where the V3 region was replaced by that of subtype B. **c** Effect of the replacement of the V3 region from isolate G in Env B and of a V3 from B in Env G. n varies between 4 and 5, depending on the sample considered

Given the dramatic impact of the polymorphism at position 202 on the functionality of Env, we focused on this position to characterize the involvement of C2 in coevolution networks through the search of amino acids that can regulate the functionality of Env through covariation with position 202. For this, we focused primarily on the case where the effect was the most marked: Env B containing the polymorphism of Env G at position 202. We therefore systematically replaced in that envelope various regions of isolate B by the corresponding regions of the G isolate, as indicated in Fig. 5a. The rationale was that if amino acids that had coevolved with position 202 were harboured in the region that was replaced functionality should have been restored. With the exception of V3, the replacement of all the other regions did not affect the functionality that remained at nearly undetectable levels. Replacing V3, instead, restored significantly (p < 0.01) the functionality of the protein to approximately 65 % that of wt B Env (Env B 202G-V3G, Fig. 5a). Therefore, although other portions than V3 probably participate to the network as suggested by the incomplete recovery of functionality of B 202G-V3G Env, V3 is largely the main component.

In order to investigate whether also in the case of G 202B Env functionality could be restored by insertion of a V3 region concordant with residue 202, we generated G 202B-V3B, which resulted to be non-functional (Fig. 5b). This result could be due to a lack of restoration of coevolution networks but it could also be explained if the replacement of the V3 region of Env G with the region V3B was not tolerated per se. To address this issue, we constructed the G V3B chimera (Fig. 5c). As shown in the figure, this chimera was not functional supporting the second hypothesis. As a control we then constructed the B V3G chimera that was fully functional (Fig. 5c). These results suggest why restoration of functionality could be observed in the case of B 202G-V3G but not for G 202B-V3B. Worth being noted, B V3G displayed a functionality of 140 % with respect to wt B Env (Fig. 5c), so the increase in functionality of B 202G-V3G with respect to B 202G could be partly due to this effect. However the amplitude of the effect on B 202G (from undetectable levels to more than 60 %) appears non-compatible with such an explanation. Finally, also data presented in the next chapter do not support this hypothesis.

### The coevolving partners of residue 202: identification of the residues

**Fig. 6:**
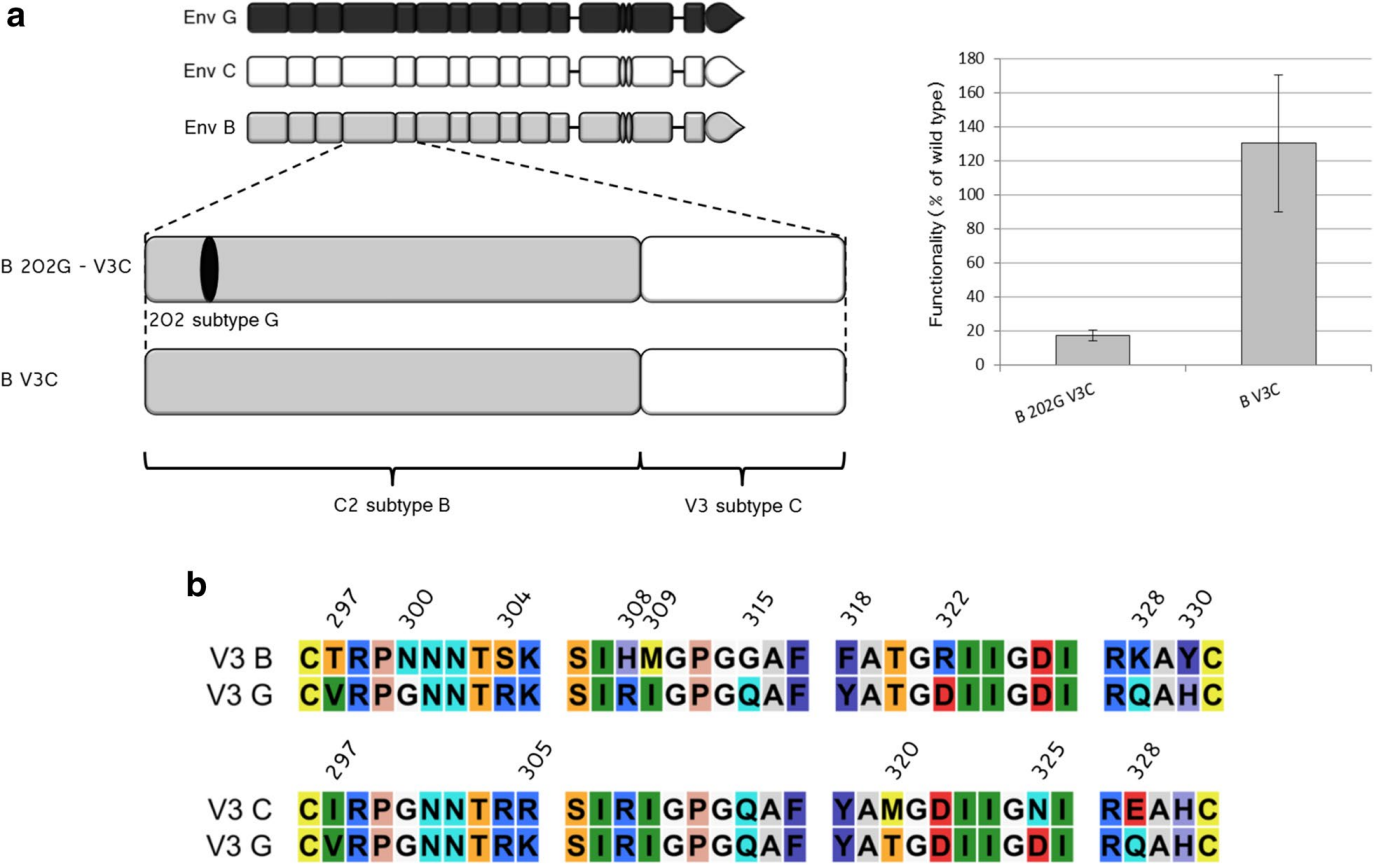
Impact of sequence divergence in V3 on the tolerance to the polymorphism at position 202. **a** *Left*, schematic drawing of B 202G-V3C and B V3C Env (wt Env used to construct these Env are given as reference for the color code, at the *top*). *Right*, functionality of B 202G-V3C and B V3C. **b** Sequence alignment of V3 of the Env B and G (*top*) and C and G (*bottom*) with annotated the 10 and 5 diverging residues, respectively (numbering is according to HxB2). Color code is the same as for Fig. 2a

The need in V3 for amino acids specifically from the G isolate in order to observe the reversion to functionality was confirmed by the replacement in B 202G of V3 by V3 from isolate C (B 202G-V3C Env, Fig. 6a) that displayed a functionality only of 18 % with respect to wt B Env. The partial recovery of functionality with respect to B 202G (18 vs <0.1 %) is likely due to the smaller number of amino acids diverging between isolates G and C than between G and B in V3 (Fig. 6b). Also in this case, we verified the tolerance of the insertion of the exogenous V3 region (in this case V3C) in wt B Env (chimera B V3C). As it was the case for B V3G, an increased functionality with respect to wt B Env was observed (130 % in this case, Fig. 6b). If the improvement of functionality in B 202G-V3G was due to the beneficial insertion of the V3 region from G, it would be expected to observe a similar increase also for B 202G-V3C. Since in this latter case the effect was instead modest (18 % of functionality with respect to 60 %) we conclude that the effect is rather due to the degree of sequence divergence between the different V3 regions.

**Fig. 7:**
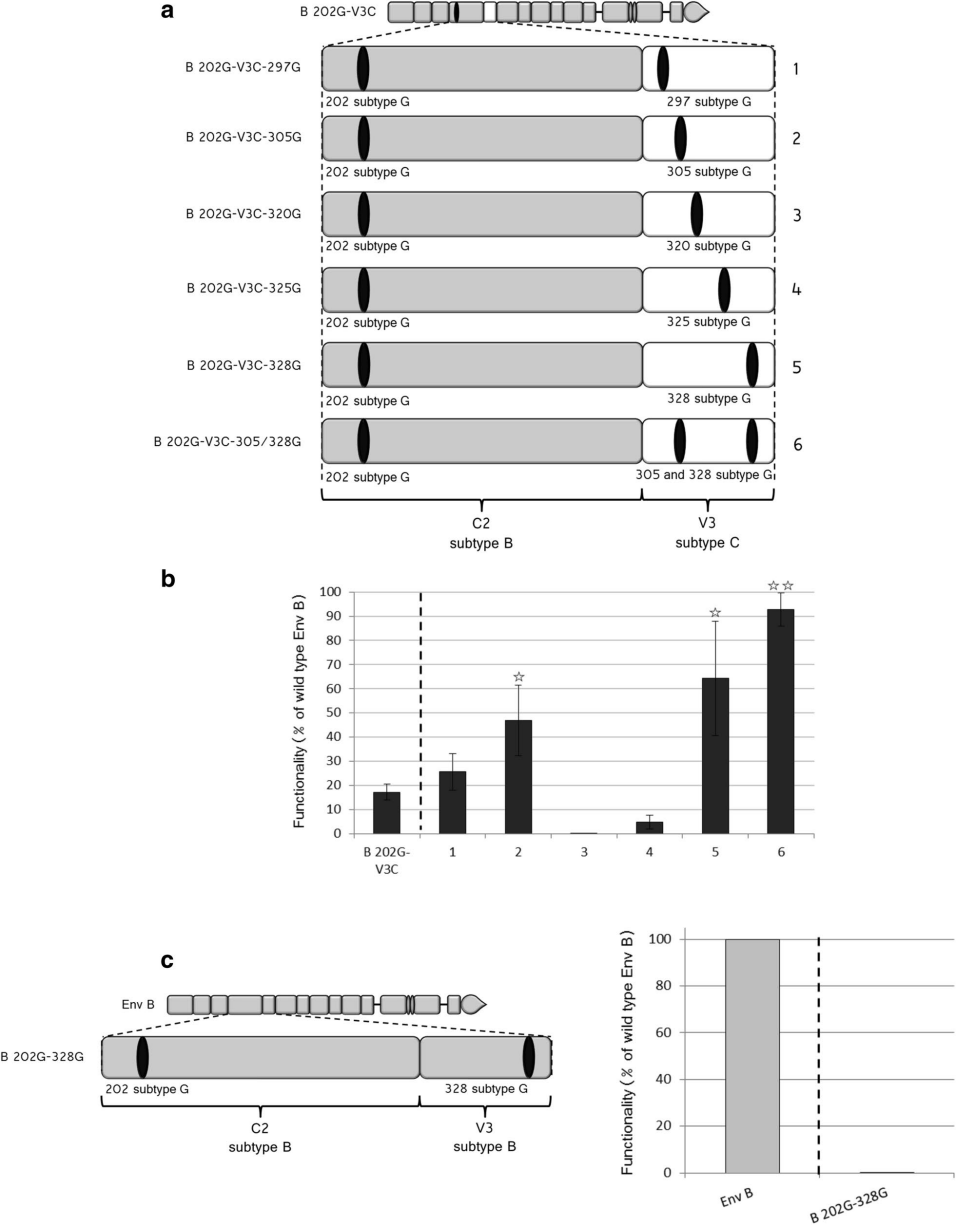
Identification of the G-specific amino acids required to restore the functionality in B 202G-V3C Env. **a** Schematic representation of the Env constructed by inserting in the V3C sequence, amino acids from the G isolate, according to the scheme. Color code: Env B *grey*, Env G *black*. **b** Functionality of the Env drawn in **a** (numbering of the samples is according to the numbering provided on the *right* of the drawings, in **a**). The value of infectivity for B 202G-V3C Env is given as reference on the *left* of the *dotted line* and the *stars* indicate that the functionality of the various Env increases significantly with respect to this reference. One star: p < 0.05; two stars, p < 0.01. Percentages of functionality were calculated as for Fig. 1. n varies between 3 and 5, depending on the sample considered. **c** Schematic drawing (*left*) and functionality (*right*) of B 202G/328G (the functionality of B 202G is given as reference on the *left* of the *dotted line*)

The lower sequence divergence between G and C in V3 was then exploited for the identification of the specific amino acids of V3 from isolate G that allow functional preservation of the Env that carries the 202G polymorphism. Indeed, the difference between V3C and V3G is limited to five positions, in contrast to the ten amino acids that differentiate G and B in V3. The identification of the specific amino acids would have been extremely difficult starting from B 202G Env, particularly considering the possibility that multiple residues might be involved simultaneously, making the number of combinations to test unrealistic. For this reason we searched the amino acids involved in the recover of functionality in B 202G-V3G starting from B 202-V3C Env. This was achieved by replacing, in B 202-V3C, individually each one of the five amino acids differing from B 202G-V3G (Fig. 7a).

As shown in Fig. 7b, the individual replacements gave contrasting results. A complete loss of functionality was observed for the replacement of the methionine at position 320 by a threonine and of an asparagine by an aspartic acid at position 325, suggesting the participation of Met 320 and of Asn 325 in coevolution networks involving other residues within V3. A significant increase in functionality, indicative of coevolution between V3 and position 202, was instead observed for the replacement of residues 305 and 328 (lysine instead of arginine and glutamine instead of aspartic acid, respectively). Functionality remained however lower than that of the original wt B Env. When the two mutations were combined (construct 6), though, the functionality of the protein was completely restored (Fig. 7b, no significant difference with respect to wt B Env, p = 0.24). This result identifies a network of residues (positions 202–305–328) that through covariation can conciliate the presence of a Lys at position 202 and a level of functionality as that of the wt Env.

Since substantial differences exist in V3 between the sequences of isolates B and C as shown in Fig. 6b, it was likely that the presence of a Lys at position 305 and a Gln at position 328, sufficient to restore functionality in B 202G-V3C, would not restore functionality in B 202G. In addition, the possibility that these two positions were not crucial for Env B was also supported by the observation that the same amino acid is found in isolates B and G at position 305 (a lysine). To verify this, we replaced the Lys (originally present in Env B at position 328) by the Gln of isolate G. No restoration of functionality was observed for B 202/328G (functionality lower than 0.01 % that of wt B, n = 3) in this case (Fig. 7c). It appears therefore that, depending on the V3 sequence, the residues allowing the restoration of functionality are not the same, reflecting functionally significant differences in the structural arrangement of this region.

### The 202 polymorphism does not affect gp120 stability on the viral particle

**Fig. 8:**
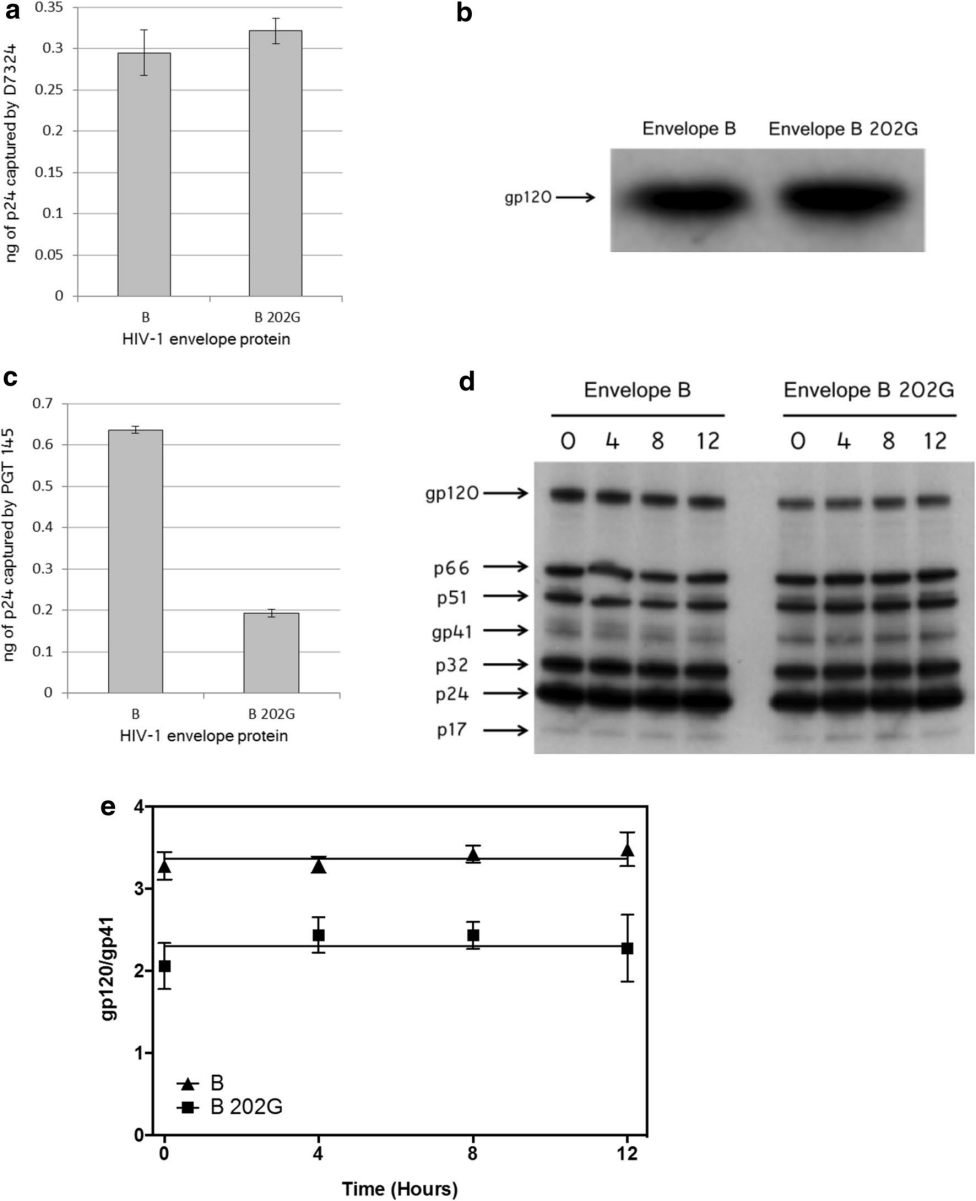
Properties of the defective B 202G Env at the surface of the viral particles. **a** Viral titer (ng of p24) estimated after retention of viral supernatant on ELISA plates coated with the antibody D7324. **b** Western blot on purified viral particles using antibody D7324. **c** Viral titer (ng of p24) estimated after retention of viral supernatant on ELISA plates coated with the antibody PGT 145. **d**, **e** Measure of the stability of the gp120 protein at the surface of the viral particle. **d** Western blot performed on purified virions carrying either wild type B or B 202G Env proteins as function of the incubation time (indicated, in hours, above each *lane*) at 37 °C. The identity of the different proteins has been inferred by their molecular mass and is indicated with *arrows*. The gel shows a representative result of three independent experiments. **e** The bands corresponding to gp120 and to gp41 in three western blots (as the one shown in **d**) have been quantified as indicated in “Methods” section and the corresponding values are expressed as gp120/gp41 ratio as function of the incubation time. *Triangles* wt B Env; *squares* B 202G Env

In order to identify at which level the perturbation of the coevolution network induced by the polymorphism at position 202 results in the abolishment of the functionality observed in B 202G we first verified the presence of the envelope in the viral particles, following a double approach: an ELISA test and a western blot analysis. For the ELISA test (also see “Methods” section) the plaque was coated with the antibody D7324, a polyclonal antibody that recognises an epitope spanning the gp120 and the gp41 subunit [33], a region that should be recognised in a similar manner in our chimeras since it is located away from the portions of proteins that differ between wt B Env and Env B 202G. D7324 was used to capture viruses from the supernatant of cells transfected to produce viral particles exactly under the same conditions as for the viral particles used for the entry tests described above. After washing the plates, the viral titer was then evaluated by measuring the amount of p24 antigen. As shown in Fig. 8a, similar amounts of protein were detected for the two samples. The same antibody was then used for the western blot analysis and, confirming the results of the ELISA test, it indicated the presence of similar amounts of Env in the two samples (Fig. 8b). Defects in the recruitment of B 202G on the viral particle are therefore not responsible for the loss of infectivity observed with B 202G Env.

Env trimer formation is essentially driven by the formation of the six-helix bundle by gp41. In our case, the gp41 component of the different Env is constant, suggesting an equivalent formation of trimers at the surface of the viral particle. However, maintain of a native, pre-CD4 bound, conformation is influenced by the nature of gp120, and this has an impact on the stability of the architecture of the whole trimer. To infer the proportion of Env in a native trimeric form formed by wt B and B 202G we made an ELISA test as the one described above, using the monoclonal antibody PGT145 that recognises a quaternary epitope present only in the native trimeric form of Env [34] This antibody was chosen because, as shown below (Table 1), it led to a similar neutralisation profile for all the chimeras tested, suggesting a similar efficiency of recognition of the various envelopes and therefore also of wt B and B 202G. This antibody appeared therefore the most promising for detecting the presence of native trimers at the surface of the viral particles. As indicated in Fig. 8c, native trimers were present for both Env tested. The approximately three times lower amount of p24 retained on the plaque for B 202G than for the wt B Env could reflect a lower proportion of native trimers for B 202G, or a worse recognition of this envelope with respect to wt B Env, or both. The detection of native trimers for B 202G indicates that this envelope should be able to lead to membrane fusion even if, in the case of a higher proportion of misfolded Env formation for B 202G, this could partly account for the decrease in functionality of this Env.

**Table 1:**
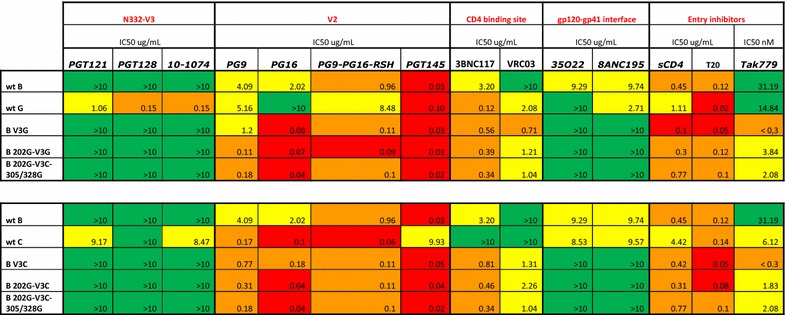
Sensitivity to antibodies and inhibitors of viral entry

Cases are coloured according to the level of IC_50_: <0.1, red; 0.1–0.99, orange; 1.0–9.99, yellow; >10.0, green. The table is divided in two groups: above, chimeras involving V3G; below, chimeras involving V3C. The values for wt B Env and B 202G-V3C-305/328G are reported as reference in both parts of the table

We then investigated whether the stability of the interaction between gp120 and gp41 was perturbed in B 202G. Decreased stability of this interaction would indeed lead to dissociation of the gp120 from the gp41 in the absence of CD4 making the viral particle unable to recognize and bind the target cell. To evaluate the stability of the gp120 at the surface of the viral particle in the absence of interaction with CD4 we incubated purified virions carrying either wt B Env or B 202G in PBS at 37 °C for different times (from 0 to 12 h) before ultracentrifugation and measuring the ratio gp120/gp41 by western blot on the pellet fraction (Fig. 8d). The rationale was that a decreased stability of the interaction between gp120 and gp41 would lead to a progressive shedding of the gp120 from the surface of the viral particle that would result in a curve with a negative slope when the gp120/gp41 ratio is plotted as a function of increasing times of incubation, as done in Fig. 8e. The flat trend of both curves, instead, indicates that both gp120 variants were stably present on the viral particles indicating that the polymorphism at position 202 does not impact the stability of the gp120 at the surface of the viral particle.

The two envelopes differed instead for the height of the curves, in Fig. 8e, which is indicative of an apparent higher gp120/gp41 ratio for wt B Env. The theoretical ratio gp120/gp41 in the viral particle is 1 and the observed ratios most likely reflect how well each of the two proteins is recognised by the serum used for the western blot. Indeed, the serum from patient better recognised gp120, which is known to be highly immunogenic, than gp41and for both Env. The difference in the heights of the curves in Fig. 8e can be explained by the possibility that the replacement of the amino acid 202 alone affect the antigenic profile of the envelope as discussed below.

### The affinity of monomeric B 202G gp120 for CD4 and CCR5 is similar to that of wt B gp120

**Fig. 9:**
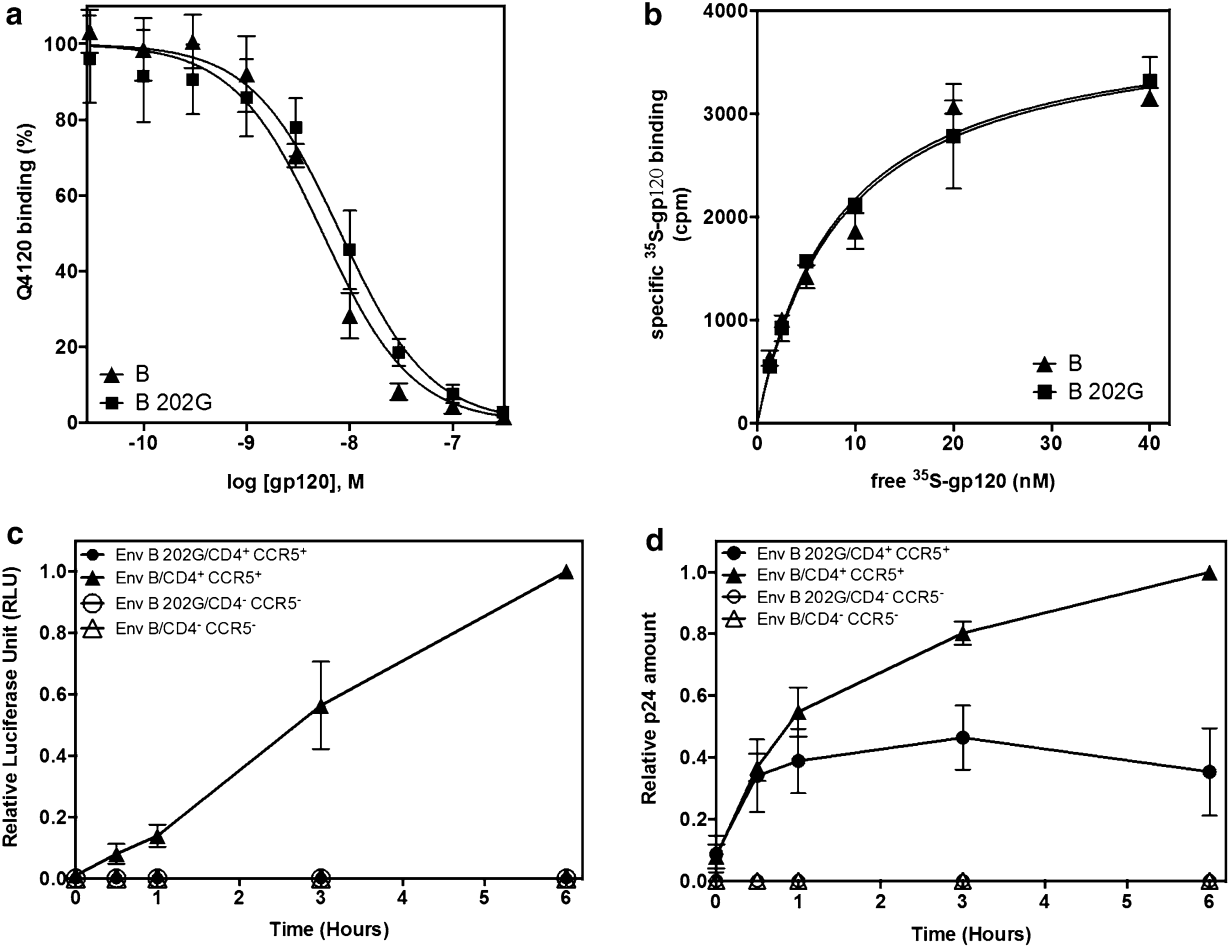
Functional characterization of the defective B 202G Env. **a**, **b** Binding efficiency of viral envelopes to CD4 and CCR5. **a** Binding competition assay at 37 °C of increasing amounts of purified soluble gp120 with 0.4 nM of the anti-CD4 mAb Q4120. *Triangles* wt B Env, *squares* B 202G Env. n = 3. **b** Equilibrium saturation binding at room temperature of wt B and B 202G gp120 to crude membranes expressing CCR5. The *curves* represent specific binding of gp120 determined in the presence of 200 nM of soluble CD4. *Triangles* wt B Env, *squares* B 202G Env. n = 3. **c**, **d** Viral binding to target cells and viral entry over time. **c** Luciferase and **d** p24 levels measured as function of the time of pre-incubation of the cells with the virions. *Filled symbols* Experiments with HEK 293T CD4^+^ CCR5^+^, *empty symbols* experiments with HEK 293T cells. *Triangles* Virions carrying wt B Env, *circles* virions carrying B 202G Env. Values measured for the 6 h time point with wt B Env and HEK 293T CD4^+^ CCR5^+^ were set to correspond to the maximum values in both *panels*. n = 3

The ability of B 202G to bind CD4 and CCR5 was then tested by a biochemical approach. Binding of purified soluble B 202G gp120 to CD4 exposed at the surface of HEK 293T cells was measured by competition with the monoclonal antibody Q4120. This antibody engages the same site at the surface of CD4 that is recognized by HIV gp120 [22] and thus sterically inhibits gp120 binding to the receptor. The equilibrium dissociation constant K_i_ value for B 202G binding to CD4 was deduced by using increasing quantities of soluble B 202G gp120 in competition with a constant quantity of Q4120 (0.5 nM). The experiment was performed in parallel with wt B gp120. Both gp120 displayed similar affinities for CD4 (K_i_ of 5.0 and 3.9 nM for B 202G and wt B gp120, respectively, Fig. 9a). This indicates that differences in CD4 binding efficiencies cannot account for the observed dramatic differences observed in viral entry with these two envelopes.

The affinity for CCR5 has then been measured by saturation binding experiments of ^35^S-labeled gp120 to purified CCR5-expressing HEK cell membranes in the presence of an excess concentration of soluble CD4 (200 nM), as previously described [35] (see “Methods” section). Specific binding of both gp120 to CCR5 could be described by hyperbolic curves (Fig. 9b) and from which we derived K_d_ values of 8.1 ± 1.7 and 8.4 ± 1.5 nM for B 202G and wt B Env, respectively. These results rule out also CCR5 binding efficiencies from the list of possible defects in functionality of B 202G Env.

### B 202G efficiently binds target cells but cannot lead to membrane fusion

To evaluate B 202G binding to CD4 in the context of the trimer expressed at the surface of the viral particle, virions encoding the firefly luciferase were added to HEK 293T CD4^+^ CCR5^+^ cells in culture and incubated at 37 °C for different times, as indicated in Fig. 9c, d. Cells were then detached mechanically by gentle pipetting and centrifuged at low speed, conditions under which free virions are found in the supernatant fraction while internalised or cell-bound virions are associated to the cell pellet (also see “Methods” section). The supernatant was discarded and the cell pellet was gently resuspended and divided into two aliquots. One was used to evaluate viral entry by measuring the expression of the luciferase (Fig. 9c), while the other was used to measure the amount of viral particles associated to the cells (those internalised plus those simply bound to them) through estimating the quantity of p24 associated to the cells (Fig. 9d).

While a progressive increase of viral entry in the target cell was observed with wt B Env for increasing times of incubation of the cells with the viruses, no entry was observed for B 202G Env irrespective of the incubation time, confirming the total lack of functionality of this Env (Fig. 8a). However, despite its lack of functionality B 202G retained the ability to bind the target cells, as indicated by the amount of p24 associated to the cells (Fig. 8b). No signal of p24 was observed for both Env on the same cells deprived of CD4 and CCR5, indicating the specific nature of the binding signal observed.

This result confirms, in the context of the trimeric Env presented at the surface of the viral particle, the observations made for the binding of the soluble gp120 to CD4, further supporting the conclusion that B 202G possess an ability to bind the receptor comparable to that of the original wt B protein. Binding of B 202G rapidly reaches a plateau, likely as a consequence of non-productive binding of this Env. For wt B Env, the time course curve expectedly paralleled the luciferase curve shown in panel A, reflecting progressive viral entry for increasing times of incubation of the cells with the viruses.

### Revertant Env has an altered antigenic profile and a different sensitivity to the entry inhibitor Tak779

In order to probe the structural arrangement of the wt Env B and of the B 202-V3C-305/328G revertant we tested their immuno- and pharmaco-properties (Table 1). Differences in the recognition by different antibodies or in the efficiency of specific inhibitors of viral entry would be indicative of differences in the conformation of the various envelopes tested. Neutralisation sensitivity to antibodies directed against V3, V2, the gp120/gp41 interface and the CD4 binding sites (CD4bs) as well as sensitivity to the entry inhibitors soluble CD4 (sCD4), T20 and Tak779) was tested. Concerning the antibodies directed against V3 a surprising observation is that the antigenic properties of V3 from isolates G or C are not retained when these V3 are inserted in the Env B backbone, rather displaying the properties of wt B Env.

This is true not only for the revertant, but also for all the other chimeras tested where V3 was exogenous (B V3G, B V3C, B 202G-V3G, B 202G-V3C) indicating that the conformation of the V3 domain is modulated by the sequence of the rest of the protein. Concerning the antibodies directed against V2 and against the CD4bs, the insertion of the exogenous V3 triggers a change in the parameters of recognition by these two classes of antibodies, in all cases leading to an increased sensitivity with respect to the starting Env (wt B Env). This is indicative of the influence of V3 on the conformation of V2 and of the accessibility of the CD4 binding site. Noteworthy, recognition of V2 by PG9 is different between B 202G-V3G and BV3G, indicating a modulation of the conformation of V2 by residue 202, either direct or through V3. In the case of PGT145, instead no increased sensitivity was observed and all Env were neutralised with similar efficiency justifying its use for the ELISA tests aiming at the estimation of the amount of trimeric Env mentioned above. Expectedly, no significant alteration of the recognition pattern was observed among the various Env for the antibodies targeting the gp120/gp41 interface, confirming that this interface was not altered in the variants of Env considered in this work. This result is in accord with the absence of major differences in the sensitivity to the entry inhibitor T20. For the other two entry inhibitors tested, the similarity of sensitivity to sCD4 suggests that all Env undergo similar transitional dynamics between different opening states of the Env trimers at the surface of the viral particle [36] and is indicative of similar affinities of the various Env for CD4. Finally, sensitivity to Tak779, an allosteric antagonist of CCR5, highlights a two-log decrease from wt B Env and B V3G, documenting the occurrence of a conformational change induced by the exogenous V3. Similarly the ten-fold difference observed with Tak779 between B V3G and B 202G-V3G shows that the introduction of the 202G polymorphism also induces a marked conformational change of V3. Overall the results obtained with Tak779 in combination with the biochemical data indicate that the mode of binding to CCR5 differs between wt B Env and the revertant B 202G-V3C-305/328G and that the return to functionality is paralleled by conformational changes in V3.

## Discussion

Coevolution is essential for allowing sequence diversification of proteins, a process particularly important for pathogens that are exposed to the immune response of the host. We report that, despite its overall conservation across HIV-1 clades, replacing the constant region C2 of HIV-1 Env of a primary isolate of subtype A or B by that of an isolate from subtype G or from subtype C triggered a dramatic decrease in functionality with respect to the wild type proteins (Fig. 1c). We focused in particular on the chimeric subtype B isolate where the C2 sequence from isolate G was inserted, since the effect was the most manifest in that case. For this chimera we identified a single polymorphism to be responsible for the total loss of functionality observed when the whole C2 region was exchanged. In particular, replacement of the Thr at position 202 (conserved in subtype B at 96.4 %) by a Lys (conserved at 96.8 % in subtype G) resulted to be sufficient to drop Env functionality to background levels. Functionality of Env B mutated at position 202 was then restored by the insertion of the V3 region from the G isolate, indicating that the coevolving partners of residue 202 were located in V3. In particular, we could identify two residues (positions 305 and 328) that, when of G origin, where sufficient to fully restore functionality (Fig. 7b). Amino acids 305 and 328 are located in the top portion of the ascending part (aa 305) and at the base (aa 328) of the V3 loop, respectively. It has been postulated by bioinformatics approaches the existence of a coevolution link within V3 between residue 328 and positions 306 and 307 [37]. Even if in our case positions 306 and 307 are not involved, it is possible that covariation between position 328 and amino acid 305, adjacent to 306 and 307, is required to provide an appropriate folding of V3. This would “link” positions 328 and 305, requiring their simultaneous replacement in the revertant.

The existence of epistatic interactions between V3 and other portions of the protein had been suggested previously, specifically in the context of the determination of coreceptor use. Indeed, even if the choice of the coreceptor is mostly dictated by interactions with V3, mutations present in the gp41, but also in C1, C2 and C4 alter this choice [38–42]. With respect to those observations that report subtle modifications of the activity of the Env, we report here for the first time that the epistatic interaction between V3 and C2 modulates the transition from completely non-functional to fully functional forms, without impacting coreceptor usage.

The restoration of a marked defect due to a polymorphism in region C2 by covariation of residues in the V3 loop connects functionally two structural elements of the protein that accumulate mutations at different paces. The lower sequence diversity present in constant regions, despite their tendency to induce higher mutation rates than variable regions [43], is intuitively attributable to more strict structural constraints that result in stronger purifying selection in these regions. By documenting the existence of coevolution between constant and variable regions, the present work shows that functional constraints acting on constant regions can influence sequence diversity in the variable ones. Conversely, the structural flexibility of the V3 region increases the genetic robustness of the protein by allowing to compensate for the negative impact of polymorphisms introduced in constant regions and, consequently, to explore a larger sequence space. Disordered regions have been previously reported to increase the sequence plasticity of their respective proteins in a series of organisms [44–46]. Although variable regions of Env cannot be considered as disordered domains and a defined architectural arrangement has been assessed for V1V2 and V3 [47, 48], their genetic diversity underscores a higher flexibility than that of constrained constant portions of the protein. The results we have obtained on V3, suggest that variable regions of Env, could play a function similar to that of disordered parts of proteins, and their presence could increase genetic robustness, besides the well-recognised role they have in the escape from the immune response.

The non-functional Env (B 202G) was neither affected in the stability of the interaction between gp120 and gp41as judged by western blot on the purified viral particles (Fig. 8d, e), nor in the ability to bind CD4, or CCR5, as shown by biochemical and cell culture data (Fig. 9a, b). Since PGT145 recognizes less efficiently B 202G than wt B Env (Fig. 8c) it is possible that the mutant yields a lower proportion of native trimers at the surface of the viral particles. This can therefore account for part of the decreased efficiency of entry by B 202G, since a correlation between number of trimeric spikes present and efficiency of viral entry has been described [49, 50]. The total absence of functionality observed, though, is indicative of the existence of an additional, strong block in the entry process.

A second clear defect triggered by the 202 polymorphism was observed downstream co-receptor binding. Biochemical tests indicate, in fact, that B 202G binds CCR5 with an affinity identical to wt B Env despite a total inability to carry out membrane fusion. Since the gp41 portion of B 202G is the same as in wt B Env, failure to carry out membrane fusion by gp41 per se can be ruled out. The defect rather seems to come from the conformational changes that lead from the CCR5-bound structure to the membrane-fusion competent one. The participation of residue 202 in the formation of the bridging sheet, which is involved in the interaction with CCR5, supports this possibility. This interpretation is also compatible with our biochemical data and with the time-course experiments of viral entry into target cells and capture of the viruses at their surface (Fig. 9) that show that, albeit B 202G does not allow viral entry, it retains the ability to bind target cells.

**Fig. 10:**
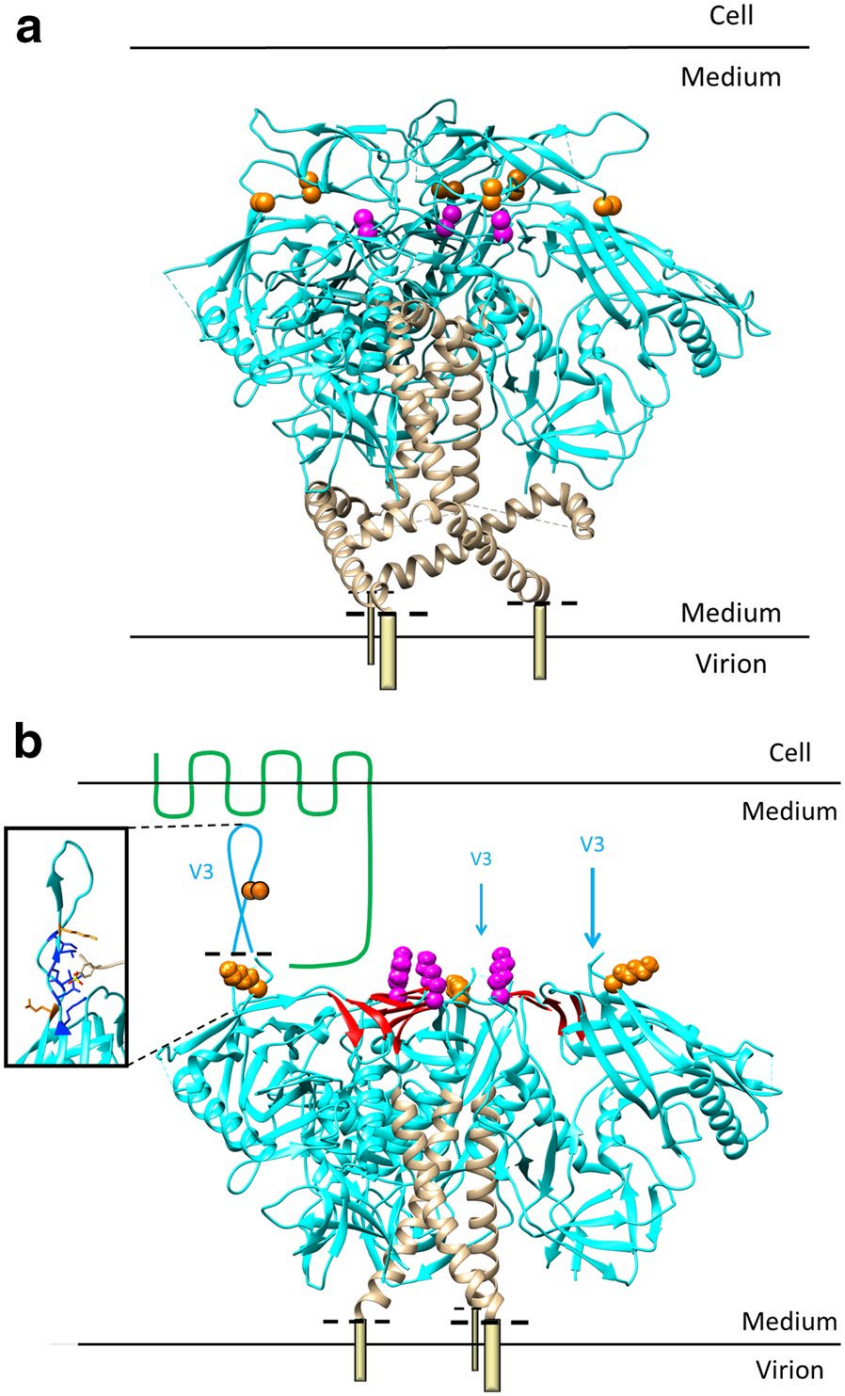
Structural modeling of the covariation network in the revertant Env. **a** Mapping amino acids 202, 305, and 328 on the structure of the crystallographic structure of HIV-1 Env, obtained on cleaved gp140 SOSIP trimers by Julien et al. [17]. gp120, *blue*; gp41, bronze with the transmembrane portion schematically drawn as a *cylinder*. *Dotted black lines* indicate the border between the structure determined in Ref. [17] and the parts drawn by us. *Orange spheres* indicate amino acids 305 and 328, *purple spheres* give the location of amino acid 202. **b** Mapping amino acids 202, 305, and 328 on the structure of the HIV-1 Env, obtained by Cryo-EM on cleaved gp140 SOSIP trimers by Bartesaghi et al. [19]. gp120 *blue*, bridging sheet *red*; gp41, bronze with the transmembrane portion schematically drawn as a *cylinder*. *Dotted black lines* indicate the border between the structure determined in Ref. [19] and the parts drawn by us. The putative folding of the V3 stem is drawn in *blue* for one monomer only, for sake of clarity (the positions of the two other V3 stems are indicated with *blue arrows*). *Orange spheres* indicate amino acids 305 (middle of the V3 stem) and 328 (base of the V3 stem), *purple spheres* give the location of amino acid 202. One molecule of CCR5 is schematically drawn in *green* (not in scale). Cell and viral membranes are schematically drawn as *black lines*. In the *black box* is depicted the docking of the sulfur-tyrosine of 412d antibody (mimicking CCR5) in the V3 loop as described by Huang et al. [23]. 412d is drawn in bronze with the sulfur in *yellow* and the oxygen atoms in *red*. The cage of lateral chains from the amino acids of V3 that surround and bind the sulfur-tyrosine is highlighted in *deep blue*. Amino acids 305 and 328 are highlighted in *yellow*

In order to evaluate the potential impact of the polymorhisms at positions 202, 305 and 328 on the conformation of the native trimers, the positions of these residues was mapped on the structure of the SOSIP Env trimer from Julien et al. [17]. In this structure, these residues do not appear to interact directly (Fig. 10a), supporting the hypothesis that the amino acid 202 coevolves with the conformation of the V3 loop as a whole, rather than specifically with residues 305 and 328, potentially explaining the absence of restoration observed for the chimera B 202/328G. Noteworthy, if coevolution occurs with the global folding of a large domain rather than with single specific amino acids, the panel of solutions to retain coevolution networks is expected to be broader and more flexible. Another important element emerging from this analysis is that residue 202 is located just beneath V1V2, which have been described to be involved in the stabilisation of the native conformation of trimers through their interaction with V3 [51]. A mutation occurring in 202 could therefore impact on the correct folding and on the stability of the trimer.

For structural considerations about the post CCR5 binding defect, the appropriate structure to refer to would be a trimeric HIV-1 Env in complex with CD4 and of CCR5. Such structure not being available, we mapped amino acids 202, 305 and 328 on the CD4-bound trimer structure [19]. In the CD4-bound state the V3 loops project away from the protein core [2, 20, 21, 52] and, consequently, a spatial proximity between residue 202 and its coevolving partners, 305 and 328 does not exist even in this structure. However, the independence of these three amino acids is only apparent here, since these positions could be bridged by a bound CCR5 molecule, as schematically indicated in Fig. 10b. Indeed, CCR5 interacts with the top and the base of the V3 loop as well as the bridging sheet and the three amino acids we describe are located in these regions. The observation that the non-functional B 202G mutant retains an unaltered ability to bind CCR5 (Fig. 9b), can be explained on the basis of the structure of the soluble CD4-gp120 monomer bound to the tyrosine-sulfated antibody 412d that mimics CCR5 binding [23]. This structure indeed shows the existence of a binding pocket for the sulphur-tyrosine of CCR5 in V3. Amino acids 305 and 328 do not participate to the formation of this binding pocket but precisely encompass it (Fig. 10b) explaining why polymorphisms in these positions do not alter the efficiency of CCR5 binding. The most plausible explanation for the loss of functionality of B 202G Env is that the arrangement of the complex Env-CD4-CCR5 formed specifically with the mutant interferes with the appropriate subsequent conformational changes that lead to membrane fusion [5]. How compensation of polymorphism 202 by the polymorphisms 305–328 occurs constitutes an interesting issue for understanding the conformational rearrangements occurring after CCR5 binding.

Finally, comparative immunological and pharmacological probing of wt B and revertant Env confirm the dramatic impact on the conformation of the whole gp120 induced by the simple polymorphisms 202. This is reflected, on one hand, by the tenfold change in the sensitivity to Tak779 observed between B V3G and B 202-V3G. On the other hand, also the antigenic properties of the protein appear strongly modulated. A major observation in this sense is constituted by the change of the recognition of antibodies directed against the V3 domain exerted by the backbone in which V3 is inserted. Similarly, immune recognition of V2 is modulated by the nature of V3. Overall, these observations underline the level of complexity of the requirements for an efficient recognition by the immune system, at least for these regions of Env. Finally, these analyses document the differences in the antigenic properties of the protein observed when transitioning from the wt B Env to the non-functional B 202G variant and, finally to the functional revertant triple mutant (B 202G-V3C-305/328G). The new functional variant, possessing a different antigenic profile broadens the repertoire of infectious variants the immune system has to deal with. Altogether, these observations shed light on a new level of complexity of the interplay between the virus and its host.

## Conclusion

Demonstrating the ability of a variable region of HIV-1 Env to rescue the deleterious effect of a genetic polymorphism in a more structurally constrained region, this work sheds light on a new aspect of the implication of variable regions of Env in the increase of genetic flexibility of this protein. This flexibility allows to generate alternative functional envelopes with new antigenic profiles, widening the repertoire of variants the immune system has to deal with.

## Methods

### Cells

HEK 293T, HEK 293T CD4^+^, HEK 293T CCR5^+^ and HEK 293T CD4^+^ CCR5^+^ were cultured in Dulbecco’s Modified Eagle Medium supplemented with 10 % foetal calf serum, penicillin (100 UI/mL) and streptomycin (100 µg/mL) (Invitrogen, Carlsbad, NM, USA) at 37 °C in 5 % CO_2_.

### Plasmids, viral sequences and alignments

The envelope coding sequences have been inserted into the commercial plasmid pcDNA 3.1D/V5-His-TOPO (Invitrogen, Thermo Fisher Scientific, Waltham, Massachusetts, USA) and were co-transfected with pNL4-3.Luc.E^−^ plasmid [53] in order to produce viral particles. The envelope-coding sequences originated from primary isolates belonging to group M. In detail, the following isolates were used: one isolate belonging to subtype A1 (GenBank accession number AF407156, referred herein as Env A); one isolate belonging to subtype B (GenBank accession number AY835448, referred herein as Env B); one isolate belonging to subtype C (GenBank accession number DQ435683, referred herein as Env C); one isolate belonging to subtype G (G-548, from Ref. [29], an isolate cloned in the laboratory from a pool of cDNA kindly provided by Dr. Martine Peeters, issued from the study described in Njai et al. [54], referred herein as Env G). These four envelope genes were used to generate all the variants of envelope described in the result section. Sequences were aligned using Unipro Ugene (http://ugene.net) for generating sequence logos and for entropy analyses, and using CLC Sequence Viewer (http://www.clcbio.com/products/clc-sequence-).

### Construction of chimerical envelopes

Chimerical envelopes between different primary isolates were constructed through overlapping PCR as previously described [16, 27, 28]. Briefly, each fragment, of a given phylogenetic origin, used to constitute the final chimeric genes was amplified independently. Primers used for these PCRs carry short complementary sequences in their extremities that can allow the different fragments to hybridize. Individual fragments produced containing the overlapping sequences are then mixed in a subsequent PCR where primers are added only after few cycles. Full-length chimera is reconstituted and amplified by adding external primers.

A similar protocol has been used to generate point mutants except that the desired mutations were directly inserted in the desired sequence by amplification with a forward and a reverse complementary primer. The forward primer was used for PCR amplification with a reverse complementary primer annealing at the 3′ end of the Env-coding sequence, while the reverse complementary primer was used for amplification with a forward primer annealing at the 5′ end of the Env-coding sequence. These PCR products were then assembled to generate the full-length gene as described above for the chimerical envelopes. All constructs were verified by sequencing.

### Production of HIV-1 particles and viral entry assay

The day before transfection HEK 293T cells were plated in 6 well plates, at a density of 8.4 × 10^5^ cells per well. HIV-1 viral particles carrying the envelopes to test and containing the luciferase gene have been produced by cotransfection of HEK 293T with pNL4-3.Luc.E- and pcDNA3.1D.Env plasmids using the polyethylenimine (PEI) method according to manufacturer’s instructions (PEI MW 25,000, linear; Polysciences, Warrington, PA, USA). Seventy-two hours after transfection, the recovered medium was filtered and the viral particles were quantified by ELISA directed against p24 capsid protein according to the manufacturer’s protocol (Innotest HIV Antigen mAb, Innogenetics, Gent, Belgium). The incorporation of equivalent amounts of the different mutants of envelope proteins in the viral particles was tested by western blot.

Transduction of HEK 293T CD4^+^ CCR5^+^ with viral particles was performed by adding 25 ng of p24 protein (corresponding to 1.5 × 10^8^ HIV virions) to 1.25 × 10^5^ cells in 24-well plates. Transduced cells were incubated at 37 °C and harvested 48 h in order to proceed to a luciferase assay by following the instructions (Luciferase Assay System, Promega, Madison, Wisconsin, USA). Luminous signal produced by luciferase have been quantified with a Glomax luminometer (Promega, Fitchburg, WI, USA). Negative controls were constituted by virions produced by cotransfection with pNL4-3.Luc.E- and pcDNA 3.1D/V5-His Topo plasmids not carrying any envelope sequence, while positive controls were constituted by virions carrying the highly functional T-ADA strains envelope.

### Statistical tests

When a mean has been compared to a reference value, as for the comparison of the functionality of a chimera with respect to the functionality of a wt reference protein (which was set at 100 %), a Student’s one sample *t* test is used. When two means (for example of two chimeras) must be compared, a Fisher-Snedecor’s F-test is first used to test the hypothesis of the equality of variances. If F-test rejects the hypothesis of the equality of variances, then a Welch’s t-test is used to test the independency of the means, otherwise a Student’s two samples t-test is used to test the independency of the means.

### Shannon entropy calculations

Clustering of amino acid sequence variation in the C2 region of HIV‑1 Env was evaluated by calculating Shannon entropy using the software provided by Los Alamos National Laboratory (LANL) (http://www.hiv.lanl.gov/content/sequence/ENTROPY/entropy_one.html). HIV-1 sequences used for this study have been downloaded from the LANL database. Sequences have been sorted by subtype (A1, B, C and G). For each subtype all available sequences were downloaded after having selected the option provided by the LANL website for random choices of only one sequences per patient. The regions coding for C2 have then been translated into protein that have been subsequently aligned separately and the alignments verified manually. In all cases, the alignments made by the software have been judged satisfying and no manual corrections have been applied. Alignments have finally been uploaded onto the LANL online software for the Shannon entropy test.

### ELISA

Cells have been transfected as described in upper section in order to produce HIV-1 viral particles. Two days post-transfection, supernatants have been harvested, filtered, and virions have been quantified by ELISA directed against p24 according to the manufacturer’s instructions (Innotest HIV Antigen mAb, Innogenetics, Gent, Belgium). Separately, 96 well-flat bottom microlon 600 plates (Greiner Bio-one GmbH, Frickenhausen, Germany) have been incubated overnight at 4 °C with 50 µL of capture antibody at 5 µg/mL in carbonate-bicarbonate solution (Sigma-Aldrich, St Louis, Missouri, USA). The capture antibodies were either sheep polyclonal antibodies D7324 (Aalto Bio Reagents, Dublin, Ireland), which are raised against the same continuous epitope APTKAKRRVVQREKR and purified for their affinity toward this epitope, or human monoclonal antibodies PGT 145 (kind gift of Martine Braibant) [34]. After incubation, plates have been washed 5 times with PBS + Tween (0.5 %) and blocked with 200 µL of PBS supplemented with non-fat milk (7 %) for 4 h at room temperature. After a second washing step identical to the first one, virions produced by transfection have been added to each well (100 ng of p24 for each well) and plates have been incubated for 2 h at 37 °C. Plates have been washed again and, to harvest the content of each well, 100 µL of RIPA have been added to each well. Plates have been incubated for 5 min at 4 °C allow a complete lysis. Content of each well have then been taken, diluted at 1/5 in PBS and an ELISA test directed against p24 has been performed according to the manufacturer’s instruction (Innotest HIV Antigen mAb, Innogenetics, Gent, Belgium).

### Western blot with primary antibody D7324

Supernatants containing viral proteins were collected for western blot analysis. Samples were then normalised in order to contain 30 ng of p24 viral proteins and were separated by electrophoresis in a 4–12 % NuPAGE Bis–Tris gel (NuPAGE Novex, Thermo Fisher Scientific, MA, USA). Proteins were transferred to an Immobilon-P polyvinylidene difluoride (PVDF) membrane (Merck-Millipore, Billerica, Massachusetts, USA) and incubated with antibody D7324. Secondary antibody recognition was performed by incubation with monoclonal anti-sheep donkey antibodies conjugated to the horseradish peroxidase (Sigma-Aldrich, St Louis, Missouri, USA). Immunoblots were revealed by a luminol based enhanced chemiluminescence substrat (Pierce ECL, Life Technologies, Thermo Fischer Scientific, Waltham, Massachusetts, USA).

### gp120 and gp41 interaction assay

The evaluation of the stability of the interaction between gp120 and gp41 was assessed by western blot. Viral particles were produced by transfection as described above. Twenty-four hours after transfection, the supernatant of the culture was filtered and viral particles were purified by ultracentrifugation on 20 % sucrose cushion, as previously described [29]. Virions were then incubated at 37 °C in PBS for 0, 4, 8 and 12 h. For each time point, samples were collected by centrifugation at 100,000×*g* for 2 h. Supernatants containing free gp120 were discarded while the viral particles present in the pellet fraction were lysed in RIPA buffer (1× PBS, 1 % NP-40, 0.5 % sodium deoxycholate, 0.05 % SDS). Samples were then incubated for 15 min at 4 °C and centrifuged at 12,000×*g* for 20 min. Supernatants containing viral proteins were collected for western blot analysis by using the same protocol described in upper sections. The only difference is that primary antibodies that have been used consist in a pool of sera of 2 patients directed against subtypes B and CRF-02 viruses respectively (kind gift of Jean-Christophe Plantier, Rouen, France). Secondary antibody recognition was performed by incubation with monoclonal goat antibodies conjugated to the horseradish peroxidase (Sigma-Aldrich, St Louis, Missouri, USA). The amount of proteins was quantified using Image Lab software (Bio-Rad, Hercules, California, USA) based on the intensity of the signal obtained for gp120 and gp41 relative to the p24 protein.

### CD4 and CCR5 binding assay

Soluble gp120 was expressed and purified as previously described [35]. Increasing concentrations of soluble gp120 were bound to 5 × 10^4^ CD4-expressing HEK 293T cells in the presence of a 0.5 nM concentration of Q4120 [55]. Specific Q4120 binding was detected with Alexa Fluor 647-conjugated goat anti-mouse IgG and quantified by FACS analysis. Results were normalized for nonspecific binding (0 %) and specific binding in the absence of glycoprotein (100 %) and were fitted to a one-site competitive binding model with the software GraphPad Prism (GraphPad Software, LaJolla, California, USA), as described in Ref. [35]. The equilibrium dissociation constants K_i_ for the gp120 s were calculated from the curves according to the Cheng and Prusoff equation K_i_ = [IC_50_/(1 + L/K_D_)], where L and K_D_ represent the Q4120 concentration (0.5 nM) and the equilibrium dissociation constant of the Q4120-CD4 complex (K_D_ = 0.4 nM), respectively. For measuring affinities of gp120-CCR5 interactions, equilibrium saturation binding experiments of ^35^S-labeled gp120 to crude membranes from CCR5-expressing HEK 293T cells were obtained as described in Ref. [35] in the presence of 200 nM of soluble CD4. Specific binding curves were obtained after subtracting from total binding the non-specific binding of glycoproteins measured in the presence of 10 µM maraviroc. Data were fitted to a one-site binding model with the software GraphPad Prism (GraphPad Software, LaJolla, California, USA).

### Virion-cell binding assay

To evaluate the ability of the envelopes to bind to the target cells and to perform viral entry, virions carrying the envelope of interest (50 ng of p24) were used to infect 5 × 10^5^ HEK 293T CD4^+^ CCR5^+^. Non-specific binding of viral particles to the cells was evaluated in two ways: measuring binding of virions carrying no envelope proteins to HEK 293T CD4^+^ CCR5^+^ and measuring binding of virions carrying the Env proteins to study to HEK 293T deprived of CD4 and CCR5. The signal obtained with virions carrying no envelope proteins and HEK 293T CD4^+^ CCR5^+^ was used to substract background signal. Virions and cells were incubated at 37 °C for different times and the cells were then pelleted by centrifugation at 400*g* for 3 min. Supernatants containing free virions were discarded and the cell pellets gently resuspended in DMEM. Half of each sample was incubated in duplicate at 37 °C, 5 % CO_2_ for 48 h and used for luciferase assay. The other half was centrifuged again 3 min at 400 g, supernatants were discarded and cellular pellets were lysed with RIPA buffer using the same protocol described above. The amount of p24 protein in pellets was estimated by ELISA, as described above.

### Env-pseudotyped virus production and titration for neutralization assays

Env-pseudotyped viruses were produced as previously described [56] by cotransfecting 3 × 10^6^ 293T cells with 4 μg of each pCDNA3.1-env clone and 8 μg of pNL4.3.LUC.R_E [53] using FuGene^®^-6 transfection reagent (Promega, Madison, Wisconsin, USA). Virus stocks were harvested 72 h later, purified by filtration (0.45 μm filter) and stored as aliquots at −80 °C. Viral infectivity was monitored by infection of 1 × 10^4^ TZM-bl cells, with serial fivefold dilutions of viral supernatants in quadruplicate, in the presence of 30 μg/mL DEAE-dextran. Infection levels were determined after 48 h by measuring the luciferase activity of cell lysates using the Bright-Glo luciferase assay (Promega, Madison, Wisconsin, USA) and a Centro LB 960 luminometer (Berthold Technologies, Bad Wildbad, Germany) [57]. Wells producing relative luminescence units (RLU) >2.5 times the background were scored as positive. The TCID_50_ was calculated as described previously [58].

### Neutralization assay

Sensitivity to HuMoNAbs of the pseudotyped viruses was assessed in duplicate in TZM-bl cells, as previously described. After titration, virus stocks were diluted to 400 TCID_50_/μL. Aliquots of 50 μL were then incubated for 1 h at 37 °C with 50 μL of threefold serial dilutions of PGT121, PGT128, 10-1074, PGT145, PG9, PG16, PG9-PG16-RSH, VRC03, 3BNC117, 35O22 and 8ANC195 (10–0.0046 μg/mL). The virus-antibody mixture was then used to infect 10,000 TZM-bl cells in the presence of 30 μg/mL DEAE-dextran. Infection levels were determined after 48 h by measuring the luciferase activities of cell lysates, as described above. Results were expressed as mean values. IC_50_ values were defined as the antibody concentration required to reduce RLUs by 50 %.

### Inhibition of entry by enfuvirtide (T20), TAK779 and sCD4-183

TZM-bl cells were employed in quadruplicate to assess the sensitivity of pseudotyped viruses to ENF, TAK779 and sCD4-183 (NIH AIDS Research and Reference Reagent Program). After titration, pseudotyped virus stocks were diluted to obtain 400 TCID_50_/mL in growth medium. Aliquots of 50 µL were then incubated for 1 h at 37 °C with 50 µL of threefold serial dilutions of ENF or sCD4-183 (10–0.0046 µg/mL). The virus-inhibitor mixture was then used to infect 1 × 10^4^ TZM-bl cells in the presence of 30 µg/mL DEAE-dextran. Infection levels were determined after 48 h by measuring the luciferase activities of cell lysates. IC_50_ values were defined as the reciprocal of the inhibitor concentration required to reduce RLUs by 50 %. Results were expressed as mean values. For TAK779 inhibition, 8 × 10^3^ TZM-bl cells per well were plated the day prior infection. Cells were first treated for 1 h at 37 °C with 150 µL of three-fold serial dilutions of TAK779 (6 µM–0.3 nM) before adding 50 µL of pseudotyped viruses normalized to 400 TCID_50_/mL. 100 µL of DMEM medium supplemented with 30 µg/mL DEAE-dextran were then added to cells. Luciferase activity was measured 48 h after infection as described above. CCR5 antagonist susceptibility was expressed as maximal percent inhibition (MPI) and IC_50_ values. Results were expressed as mean values.
